# Complex *de novo* tetrasomy and trisomy of 2p22.2 involving *EIF2AK2* in a child with global developmental delay: a case report and literature review

**DOI:** 10.3389/fped.2026.1755339

**Published:** 2026-02-17

**Authors:** Jun Wang, Xin Duan, Chaolong Xu, Tianyu Song, Danmin Shen, Fang Fang

**Affiliations:** Department of Neurology, Beijing Children’s Hospital, National Center for Children’s Health, Capital Medical University, Beijing, China

**Keywords:** 2p22.2 microduplication, copy number variations, *EIF2AK2*, global developmental delay, pure tetrasomy and trisomy of 2p

## Abstract

**Background:**

While numerous copy number variations (CNVs) associated with global developmental delay (GDD) have been extensively studied, CNVs on chromosome 2p remain underreported and poorly understood, particularly those involving the *EIF2AK2* gene at 2p22.2. This study presents a novel case of pure partial tetrasomy and trisomy of 2p, advancing the understanding of genotype-phenotype correlations in this chromosomal region.

**Case presentation:**

We present a 7-year-old male who presented with GDD, primarily affecting motor and language skills. Initial symptoms included poor balance and exercise tolerance at 15 months, followed by mild dysarthria and an abnormal gait at 3 years. Physical examination revealed high-set ears, ear leakage, and flat feet. Cranial MRI indicated ventriculomegaly, hypomyelination, and white matter volume loss. Genetic analysis identified two adjacent *de novo* copy-number gains at chromosome band 2p22.2, one showing tetrasomy and the other trisomy, resulting in a complex genomic amplification involving the EIF2AK2 gene. Whole Exome Sequencing (WES) and Chromosome Analysis by Medium Coverage Whole Genome Sequencing (CMA-seq) confirmed the presence of triplication and duplication, which were not present in the proband's parents. This case highlights a rare instance of pure partial tetrasomy and trisomy 2p22.2.

**Conclusion:**

We report a complex *de novo* gain involving adjacent tetrasomy and trisomy segments at the 2p22.2 locus. Although formally classified as a Variant of Uncertain Significance (VUS) due to the lack of established dosage-sensitive genes, the involvement of *EIF2AK2* suggests a potential pathogenic mechanism. We propose that the increased genomic dosage may trigger dysregulation of the integrated stress response (ISR) via a concentration-dependent gain-of-function effect, mirroring the phenotype of pathogenic point variations.

## Introduction

Global Developmental Delay (GDD) is a term employed to describe significant Developmental Delays (DDs) observed in a child around five. Typically, this condition is characterized by a delay of at least two standard deviations below the mean in two or more areas of developmental functioning (1). Due to the wide etiology of GDD, the vast majority of cases do not have definitive causes (2). Up to forty percent of cases of DD are triggered by genetic factors. Of the genetic causes, 25% of cases are characterized by chromosomal abnormalities, for example, structural chromosomal abnormalities (3).

Copy number variations (CNVs) are considered a type of structural chromosomal abnormality that specifically refer to variations in the number of copies of a particular segment of DNA. This can involve duplications (extra copies) or deletions (loss of copies) of sections of the genome. The formation of CNVs arises from highly complex and diverse mechanisms, leading to genomic alterations such as deletions, insertions, duplications, inversions, and translocations. By analyzing the DNA sequences around breakpoints, it is possible to infer or pinpoint the causes of genomic variation and how these CNVs affect gene expression, which in turn helps to elucidate their role in causing GDD. As a growing number of case studies have identified CNVs in patients with GDD, investigating the mechanisms of CNV formation through sequencing offers a significant avenue for understanding genotype-phenotype correlations.

To date, a complex *de novo* genomic gain at 2p22.2, characterized by adjacent segments of tetrasomy and trisomy, has not been documented in patients with GDD. Here, we presented the first clinical report of a patient showing this unique type of complex structural variation. Interestingly, the duplicated region encompasses 13 genes. Among them, *EIF2AK2* (OMIM* 176871), *CRIM1* (OMIM* 606189), and *STRN* (OMIM* 614765) are recorded as disease-associated genes in the OMIM database. However, the ocular and renal phenotypes associated with *CRIM1* and *STRN* are absent in our patient. In contrast, novel pathogenic variants of the *EIF2AK2* gene have recently been reported as a new genetic cause associated with GDD. To put it briefly, the expression of the *EIF2AK2* gene plays a crucial role in determining cellular fate (4). Dysregulation of this gene, whether through excessive protein synthesis or increased apoptosis, can have profound effects on the development of organs and tissues. These effects are particularly pronounced in neurodevelopment, where such disruptions are associated with the manifestation of GDD.

Our case presents a unique instance of pure partial tetrasomy and trisomy on chromosome 2p22.2, contributing valuable insights to the existing body of literature on 2p duplications. Previous studies have largely focused on duplications in regions such as 2p11.2, 2p16.3, and 2p25.3, which are frequently associated with neurodevelopmental disorders, autism spectrum disorder, and craniofacial dysmorphisms. However, duplications affecting the 2p22.2 region, which includes the *EIF2AK2* gene, have been less extensively documented, making our findings particularly significant. Thus, our case not only expands the phenotypic variability associated with 2p duplications but also highlights the need for further research into the role of *EIF2AK2* and other genes in less-characterized regions, such as 2p22.2, in neurodevelopmental and psychiatric conditions (5).

## Case description

**Figure 1:**
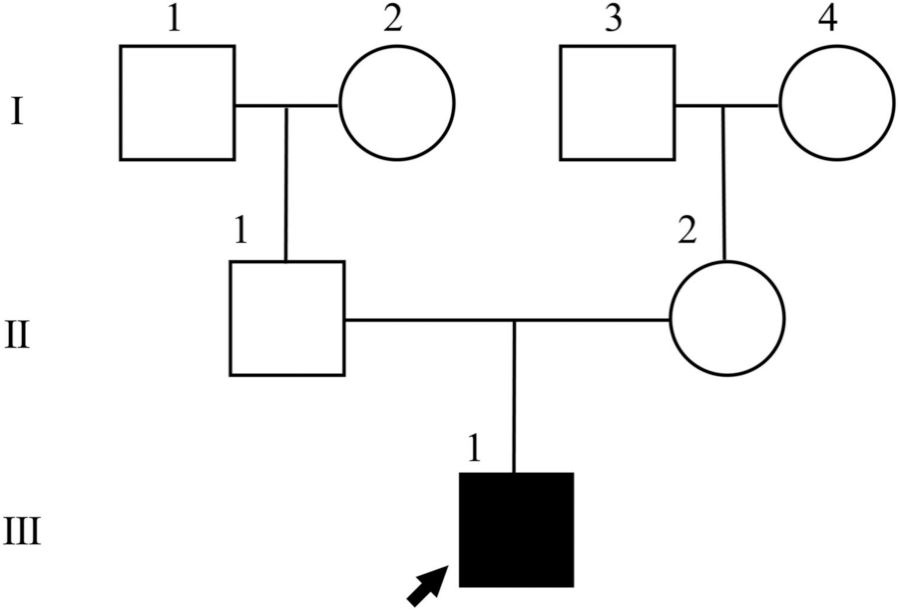
Pedigree of the family. The arrow indicates the proband (Ⅲ-1).

The proband is a 7-year-old male who presented to the Neurology Department of Beijing Children's Hospital, Capital Medical University, on November 27, 2023, due to developmental delay (DD) and an abnormal gait for over two years. He was the first child of healthy, non-consanguineous parents and was delivered at term by uncomplicated vaginal birth with a birth weight of 2.7 kg. The neonatal period was uneventful, with no history of hypoxia, jaundice, or other complications. However, intermittent episodes of bilateral purulent otorrhea were noted since birth. The neonatal hearing screening was documented as “passed” bilaterally, and no initial auditory concerns were reported by the parents. He could lift his head at 4 months and roll over at 5 months. He began walking independently and jogging at 15 months, but he was unable to jump or stand on one leg, showing poor balance and reduced exercise tolerance. At the age of 2, he began speaking, saying simple words such as “papa” and “mama”. However, his speech development was slow, and articulation was unclear. At 3 years old, his parents noticed a valgus gait in his right foot, but he rarely fell and received no treatment. At the age of 4, the patient was evaluated at a local hospital, where physical examination indicated flat feet. Cranial MRI revealed mild bilateral ventriculomegaly, periventricular T2 hyperintensity, hypomyelination with white matter volume loss in the cerebral hemispheres, and absence of normal T2 hypointensity in the posterior limb of the internal capsule. The patient had no history or signs of seizures. No episodes of neurological decompensation or new symptoms triggered by fever or other precipitating illnesses were reported. Developmental assessment revealed moderate delays in motor and language development. Rehabilitation then resulted in gradual improvements in speech, slight enhancements in gait, and improved balance. Head circumference was 47.5 cm, consistent with microcephaly. Examination of the head and neck revealed high-set ears, bilateral otorrhea, and a high nasal bridge. The skin showed no abnormal pigmentation or depigmented skin patches. The Neurological exam showed intact strength and reflexes (biceps, triceps, patellar, and Achilles) with no clonus or Babinski signs. Cardiac ultrasound was unremarkable. A developmental quotient (DQ) assessment revealed moderate motor and language delay, supporting a provisional GDD diagnosis. Blood and urine metabolic screens and ophthalmic examination were normal. Pedigree analysis (Figure 1) revealed no similar parental phenotypes.

**Figure 2:**
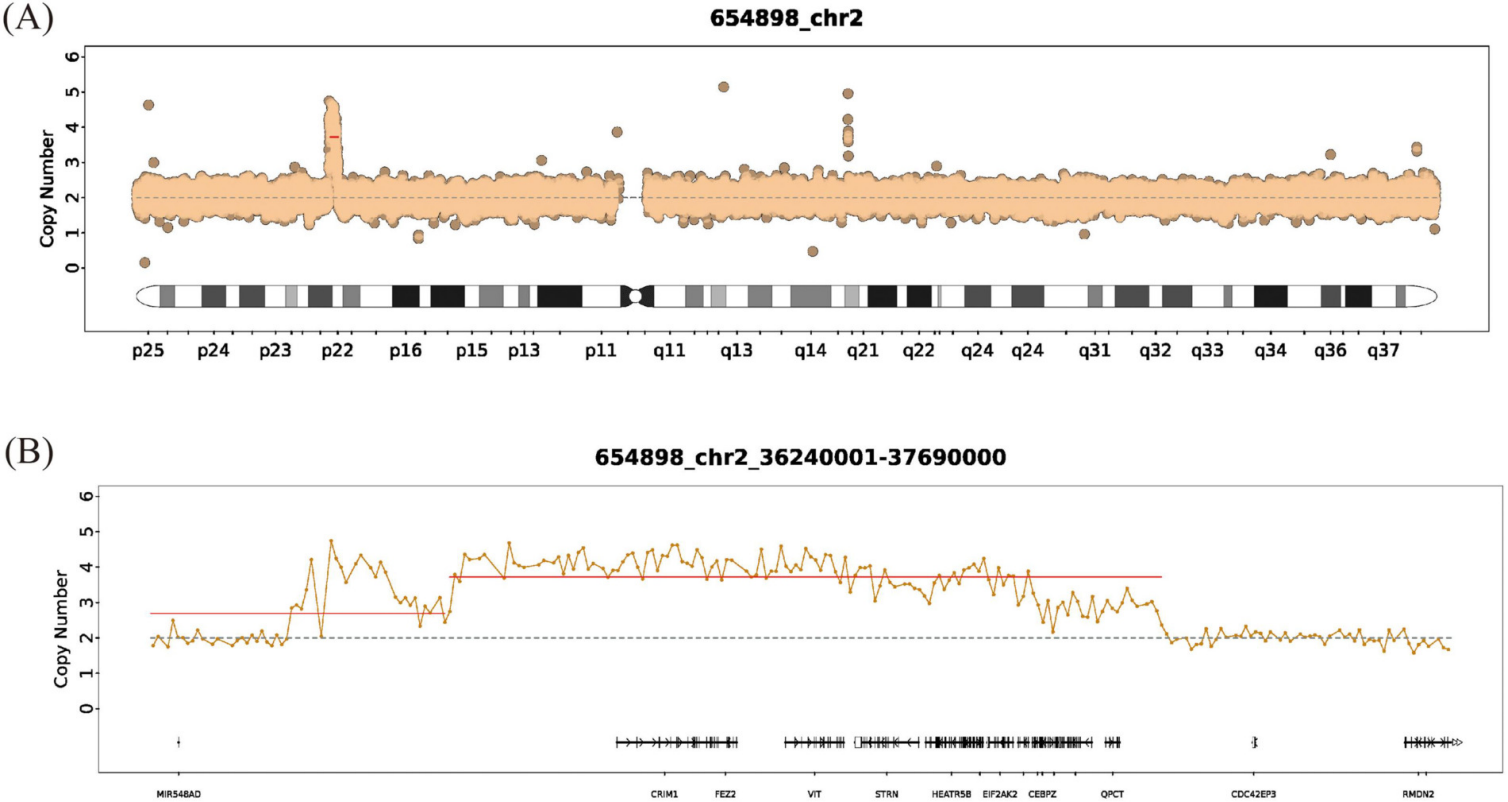
WES-based copy-number analysis of chromosome 2 in the proband. **(A)** Whole-chromosome 2 copy-number plot showing a focal gain at band 2p22.2, where normalized read depth deviates above the diploid baseline of two copies. **(B)** Zoomed-in copy-number plot of the 2p22.2 interval seq[hg19] 2p22.2 (36,240,001–37,690,000), demonstrating a complex duplication composed of a proximal segment with an estimated copy number of four and a distal segment with an estimated copy number of three, with genes in this region displayed along the *x*-axis.

**Figure 3:**
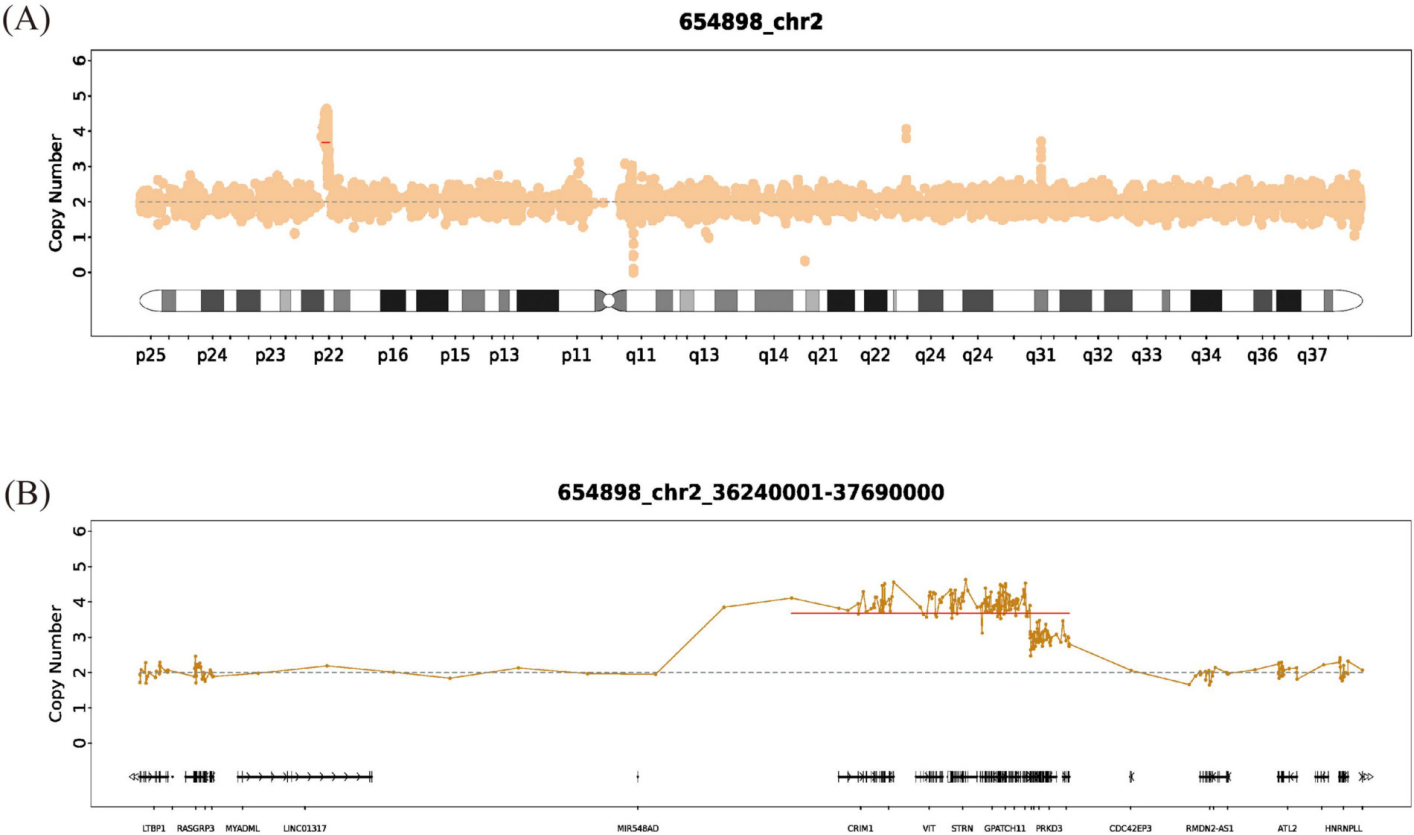
CMA-seq copy-number analysis of chromosome 2 in the proband. **(A)** Whole-chromosome 2 copy-number profile showing a focal copy-number gain at band 2p22.2, where the normalized read depth rises above the diploid baseline of two copies. **(B)** Enlarged CMA-seq copy-number plot of the 2p22.2 interval seq[hg19] 2p22.2 (36,240,001–37,690,000), demonstrating a complex duplication with an estimated copy number of three to four copies and encompassing multiple genes in this region, including *CRIM1*, *FEZ2*, *VIT*, *STRN*, *HEATR5B*, and *EIF2AK2*.

For further diagnosis, at the age of 5 years, the patient's and his parents' peripheral blood samples were collected separately for Whole Exome Sequencing (WES) (AmCare Genomics Laboratory, Guangzhou, China), along with Chromosome Analysis by Medium Coverage Whole Genome Sequencing (CMA-seq) for the patient. Genetic testing identified two novel *de novo* duplications on chromosome 2p22.2 (Figures 2, 3), spanning a total of approximately 1.45 Mb (chr2:36,240,001–37,690,000). Based on the ACMG/ClinGen guidelines, the identified 1.45 Mb complex duplication was classified as a Variant of Uncertain Significance (VUS). This classification was derived from a comprehensive evaluation of the following evidence. First, regarding population frequency, this copy number variant was absent from the Database of Genomic Variants (DGV, http://dgv.tcag.ca/). Additionally, a query of the DECIPHER database (https://www.deciphergenomics.org/) yielded no reports of similar CNVs within the specific intervals of chr2:36,240,001–37,390,000 and chr2:37,390,001–37,690,000. Second, genomic content analysis revealed that the total 1.45 Mb rearranged region consists of adjacent 1.15 Mb and 300 Kb duplication segments. This interval encompassed 13 protein-coding genes, including *GPATCH11*, *SULT6B1*, and *HEATR5B*, but crucially does not overlap with any established triplosensitive regions or genes. Third, pedigree analysis confirmed that neither parent carried this rearrangement, establishing it as a *de novo* event (Category 5A, score 0.15) originating from the maternal chromosome in the proband with no relevant family history. The first duplication involved 4 copies spanning 1.15 Mb (chr2:36,240,001–37,390,000), and the second involved 3 copies spanning 300 Kb (chr2:37,390,001–37,690,000). CMA-seq analysis further refined the breakpoints, identifying three distinct duplication regions with zygosities of ×4.08, ×3.57, and ×2.85. Among the 13 genes within the duplicated interval, *EIF2AK2, CRIM1, and STRN are* recored as pathogenic genes in the OMIM database. *CRIM1* and *STRN* are primarily associated with features not prominent in our patient as ocular, renal, or cardiac phenotypes. *EIF2AK2* has been implicated in brain white matter disorders, DD, neurodegenerative syndromes, and dystonia type 33 (6), making it the most plausible candidate gene driving the neurodevelopmental phenotype. At the age of 6 years, the patient returned for a follow-up visit. His language development laged behind that of his peers. Despite showing a mild hemiplegic gait with the right lower limb in an abducted position during walking, he was able to climb stairs independently. The muscle tone of other limbs remained normal. No Reflexes, including the Babinski sign, no spasticity, and no history or signs of seizures were observed. Regarding the otorrhea, no recurrent infections or further symptoms have been reported since the previous evaluation. The patient continued rehabilitation training and took muscle dystonia improvement medications if necessary, with ongoing follow-up and observation.

To better understand the clinical significance of the identified CNVs, we conducted a literature review of the past five years by searching Google Scholar and the PubMed database using the keywords Developmental Delay, *EIF2AK2*, and *de novo*. To date, 12 individuals with EIF2AK2-related neurodevelopmental disorders have been reported, including our patient (Table 1), comprising 10 males and 1 female from three independent cohorts (6–8). All the identified variations in these cases were *de novo* missense variants. Specifically, these included c. 326C > T (p.Ala109Val), c. 325G > T (p.Ala109Ser), and c. 290C > T (p.Ser97Phe), which were reported in Pt6 and Pt12, Pt7 and Pt10, and Pt9 and Pt11, respectively. According to the clinical phenotypes described in the literature, patients with *EIF2AK2*-related diseases are predominantly male and commonly present with Developmental Delay (DD), cerebral volume loss, hypotonia, spasticity, hypomyelination/abnormal myelination, hypertonia, abnormal T2W signals, seizure history, and gait ataxia. These features were reported in 10, 10, 9, 9, 9, 8, 8, 7, and 6 patients, respectively. It is noteworthy that only a few patients (Pt9, Pt11, Pt12) exhibited abnormal T1W signals. Additionally, some patients showed rare symptoms, such as urinary and fecal incontinence, silent aspiration of thin liquids (Pt2), or abnormal eye movements concerning seizures (Pt4).

**Table 1:** Molecular and clinical features of 12 patients with *de novo* EIF2AK2-related developmental delay.

Pts		Pt 1 (current case)	Pt2 (6)	Pt3 (6)	Pt4 (6)	Pt5 (6)	Pt6 (6)	Pt7 (6)	Pt8 (6)	Pt9 (6)	Pt10 (7)	Pt11(#1) (8)	Pt12(#2) (8)
Genotype		2p22.2	c.31A > C (p.Met11Leu)	c.398A > T (p.Tyr133Phe)	c.973G > A (p.Gly325Ser)	c.1382C > G (p.Ser461Cys)	c.326C > T (p.Ala109Val)	c.325G > T (p.Ala109Ser)	c.95A > G (p.Asn32Ser)	c.290C > T (p.Ser97Phe)	c.325G > T (p.Ala109Ser)	c.290C > T (p.Ser97Phe)	c.326C > T (p.Ala109Val)
Inheritance		*de novo*	*de novo*	*de novo*	*de novo*	*de novo*	*de novo*	*de novo*	*de novo*	*de novo*	*de novo*	*de novo*	*de novo*
Gender		M	M	M	F	M	M	M	M	M	M	M	M
Age		5 y	10 y	13 y	3 y	18 y	19 y	3 y	12 y	4 y	6 y	5 y	22 m
Dysarthria or nonverbal		Dysarthria	Dysarthria	Dysarthria	Dysarthria	Nonverbal	Nonverbal	Dysarthria	Dysarthria	Nonverbal	Nonverbal	Nonverbal	N/A
DD		+	+	+	+	+	+	+	+	+	+	+	+
Neurology	Ambulatory	Able to walk independently and jog but unable to jump or stand on one leg.	−	−	−	+	+	−	−	+	−	−	−
	Gait ataxia	−	+	+	+	N/A	N/A	+	−	−	+	+	+
	Dystonia	−	+	+	−	+	+	−	−	+	+	−	−
	Hypotonia	−	+	+	+	+	+	+	−	+	+	+	+
	Hypertonia	−	+	+	−	+	+	+	+	+	−	+	+
	Hyperreflexia	−	−	+	−	+	−	+	+	+	N/A	+	−
	Myoclonus	−	+	−	−	−	−	−	−	−	N/A	N/A	N/A
	Spasticity	−	−	+	+	+	−	+	+	+	+	+	+
	Seizures	−	+	−	−	+	+	−	−	+	−	+	−
	Seizure history	−	GTC	N/A	concern for seizure activity, normal EEG	focal complex seizures, focal epileptiform discharges	focal tonic seizures, multifocal epileptiform discharges, seizure onset at 7 months old	N/A	−	focal complex seizures, focal epileptiform discharges, seizure onset at 4 months old	−	seizures at the first year of life	seizures at the first year of life
	OFC at latest assessment	47.5 cm (*Z* = −1.67)	53.2 cm (*Z* = −0.05)	52.8 cm (*Z* = −0.66)	44.50 cm (*Z* = −1.18, 17 months)	43 cm (*Z* = −3.0)	44.5 cm (*Z* = −2.42)	48.8 cm (*Z* = −1.4)	49 cm (*Z* = −1.0)	49 cm (*Z* = −1.61)	43.5 (*Z* = −6)	44.5 (*Z* = −1.17)	41 (*Z* = −2.67)
	Additional features	High-set ears, bilateral otorrhea and a high nasal bridge	Urinary and fecal incontinence, silent aspiration of thin liquids	Intellectual disability, dysphagia, poor eye contact	Abnormal eye movements concerning for seizure	Acquired microcephaly, laryngomalacia, gastroparesis, head titubations	Exacerbation of epilepsy with febrile illnesses	Progressive contractures, walks in a crouched position with elbows flexed, thumbs adducted, bilateral feet pronation	Acquired microcephaly	Failure to thrive	Failure to thrive	Failure to thrive, hypertonia, hyperreflexia, bradykinesia, and dysmetria	Failure to thrive
MRI	Age at assessment	4 y	7 y	10 y	17 m	6 m	18 m	4 y	8.5 y	4 y	2 y	4 y	13 m
	Cerebral volume loss	+	+	−	+	+	+	+	+	+	+	+	+
	T1W signal	Isointense	Isointense	Isointense	Isointense	Isointense	Isointense	Isointense	Isointense	Hyperintensity throughout the supratentorial and infratentorial white matter	Isointense	Progressed myelination	Minimal progression in myelination
	T2W signal	Periventricular T2 hyperintensity, white matter volume loss in the cerebral hemispheres, and an absence of normal T2 hypointensity of the internal capsule	Hyperintensity, dorsal-most upper cervical cord, dorsal medulla, dorsal pons, periaqueductal gray	Hyperintensity, confluent signal in subcortical and periventricular white matter, patchy signal in brainstem	Isointense	Isointense	Isointense	Hyperintensity, dorsal medulla and periventricular	Hyperintensity, posterior part of putamen, periventricular and deep white matter, inferior cerebellar peduncles	Hypointensity throughout the supratentorial and infratentorial white matter	Hyperintensity of the white matter	Diffusely hypomyelinated with symmetrically hyperintense signal	Minimal progression in myelination
	Hypomyelination/abnormal myelination	+	+	+	+	N/A, age greater than 2 years	N/A, age greater than 3 years	+	+	+	+	+	+

Pt, Patient; +, Present; −, Absent; N/A, not available; y, years; m, months; M, Male; F, Female; DD, Developmental delay; T1W, T1-weighted; T2W, T2-weighted; EEG, electroencephalography.

In this study, the proband (Pt1) showed similar clinical symptoms, including DD, abnormal gait (mild hemiplegic-like gait and dysmetria), and MRI findings showing cerebral volume loss, hypomyelination/abnormal myelination, and abnormal T2W signals, which align with the majority of previously reported cases. However, Pt1 also presented with features not commonly reported in the literature, such as high-set ears, bilateral otorrhea, and a high nasal bridge. These findings suggest that *EIF2AK2* variations showed notable individual variability. Genetic analysis revealed that Pt1 harboured a CNV in the 2p22.2 region, involving the *EIF2AK2* gene, a region that is less frequently reported in the literature, while the majority of other cases involved point variations (SNVs) within the *EIF2AK2* gene. Despite differences in variation locations, all variations were *de novo*, and the clinical features were consistently associated with neurodevelopmental delays, reinforcing the link between *EIF2AK2* gene variations and developmental disorders.

## Discussion

GDD is a complex neurodevelopmental disorder with significant genetic underpinnings, particularly structural genomic alterations such as Copy Number Variations (CNVs). CNVs are segments of DNA that vary in their copy number among individuals, encompassing deletions, duplications, and other structural rearrangements (9). Although many CNVs are benign and do not affect development, a subset of CNVs involves dosage-sensitive regions that, when altered, result in pathological developmental consequences (10). Current research highlights a robust association between the size of CNVs and their pathogenic potential in GDD. Larger CNVs, especially those exceeding 500 kb, are associated with a heightened risk of developmental disorders, likely due to their impact on gene dosage and subsequent disruption of critical developmental pathways (10–12). Moreover, CNVs overlapping with regions known for established microdeletion or microduplication syndromes, such as Prader-Willi, Angelman, cat eye, and DiGeorge syndromes, are often pathogenic because of their disruption of genes essential for early human development (13, 14).

In this study, we reported a case of GDD associated with a complex *de novo* genomic gain at chromosome 2p22.2, representing the first clinical characterization of a structural rearrangement involving adjacent tetrasomy and trisomy at this locus. This CNV diverges from more commonly studied CNV loci, such as those at 1q21.1, 15q11.2, and 16p11.2, which are frequently linked to GDD and other neurodevelopmental disorders (15–17). The identified CNV spans 1.45 Mb at 2p22.2 and encompasses two contiguous duplications, suggesting a complex structural alteration. These duplications may have significant implications for gene dosage and expression, potentially perturbing neurodevelopmental pathways and contributing to the patient's GDD phenotype (14). Considering that a complex genomic configuration comprising adjacent tetrasomy and trisomy at 2p22.2 has not been previously documented in clinical literature, our findings suggest that such high-copy gains warrant consideration as a potential pathogenic mechanism for GDD.

CNVs can arise through various mechanisms involving chromosomal structural variations, such as Non-Allelic Homologous Recombination (NAHR), Fork Stalling and Template Switching (FoSTeS), Microhomology-Mediated Break-Induced Replication (MMBIR), and Non-Homologous End Joining (NHEJ). These mechanisms contribute to genomic instability, leading to deletions, duplications, and other rearrangements (18–20). In the case of our proband, the identified CNV spanning 1.45 Mb at 2p22.2 is likely to involve one or more of these mechanisms, contributing to the observed duplications and subsequent dosage imbalance of key genes in this region. The potential consequences of CNVs can include both gain-of-function and loss-of-function effects. For example, duplications may lead to a gain-of-function through increased gene dosage, resulting in excessive gene expression, whereas deletions may lead to a loss-of-function due to reduced gene dosage (21). In the case of our proband, the CNV encompasses duplications of critical genes, which likely result in a gain-of-function effect that could be detrimental to neurodevelopment, contributing to the GDD phenotype.

The CNV identified in our patient spans a genomic region encompassing 13 OMIM genes. Although *CRIM1* and *STRN* are recorded as pathogenic genes in the OMIM database and are located within this interval, *EIF2AK2* is considered the primary driver of the neurological features in this case. Notably, our patient presented with dysmorphic features (high-set ears, high nasal bridge) not typically reported in cases with *EIF2AK2* point variants. Since the 1.45 Mb duplication encompasses 12 other genes (including *GPATCH11* and *SULT6B1*), we hypothesize that these specific phenotypic features may result from a “contiguous gene effect” driven by the copy number gain of flanking genes, or represent a distinct phenotypic spectrum specific to structural variations. *EIF2AK2*, also known as PKR, encodes a serine/threonine kinase that plays a critical role in the integrated stress response (ISR) pathway by regulating cellular responses to various stressors (4). Recent studies have highlighted the role of *EIF2AK2* in neurodevelopmental disorders. However, previously reported pathogenic variants have been predominantly *de novo* missense variations acting via a gain-of-function mechanism (6). These variations typically lead to aberrant ISR activation. This activation impairs neuronal differentiation and synaptic plasticity which are essential for neurodevelopment (22). Despite the absence of direct functional data, established biochemical properties of PKR support a plausible dosage-dependent gain-of-function hypothesis. PKR activation is strictly dependent on homodimerization, a structural prerequisite for autophosphorylation and substrate recognition (23, 24). According to the law of mass action, the increased intracellular concentration of PKR monomers driven by genomic duplication theoretically increases the probability of spontaneous dimerization. Consequently, this lowers the activation threshold even in the absence of canonical stress signals like dsRNA. This concentration-dependent auto-activation has been validated in cellular models where overexpression of wild-type PKR was sufficient to induce constitutive translational repression and apoptosis (22, 25). We therefore hypothesize that the *EIF2AK2* microduplication mimics the hyperactivation phenotype of pathogenic point variations via a dosage-driven mechanism. This leads to aberrant ISR activation and subsequent neurodevelopmental deficits. This hypothesis aligns with observations of excessive apoptosis and abnormal synaptic development in EIF2AK2-related disorders (26, 27). These findings suggest that EIF2AK2 dysregulation converges on a shared pathogenic pathway regardless of whether it arises from structural genomic alterations or point variations. This reinforces the critical role of the gene in the pathogenesis of GDD.

To evaluate the clinical relevance and pathogenic potential of the *EIF2AK2* copy-number gain, we conducted a systematic query of population and clinical genomic databases. Data from the Genome Aggregation Database for Structural Variants (gnomAD-SV v4.1.0) demonstrate that gains involving *EIF2AK2* are exceptionally rare in the general population. Identified duplication events are limited to singleton or doubleton alleles (Allele Count <3 out of ∼126,000) and primarily represent partial or TSS-associated duplications rather than complete gene gains. This rarity is further corroborated by the Database of Genomic Variants (DGV), where full-gene duplications were not observed in healthy control cohorts, suggesting a genomic intolerance to increased *EIF2AK2* dosage. Computational evidence from the DECIPHER database strongly supports this dosage-sensitive profile, as *EIF2AK2* carries a high probability of triplosensitivity (*p*Triplo = 0.80) and a high likelihood of a gain-of-function mechanism (*p*GOF = 0.830). Furthermore, the DECIPHER genome browser reveals a distinct clustering of regional duplications overlapping the *EIF2AK2* locus, which are primarily associated with neurodevelopmental phenotypes such as intellectual disability and global developmental delay (GDD). Notably, we identified a matching clinical case (Patient ID: 414938) harboring an identical 1.45 Mb genomic span and presenting with GDD. While this matching case is also formally classified as a Variant of Uncertain Significance (VUS), the striking recurrence of this specific genomic architecture in individuals with shared neurodevelopmental features provides compelling support for *EIF2AK2* as a critical candidate gene contributing to the observed phenotype.

Collectively, the potential clinical relevance of this locus is supported by the convergence of several lines of evidence, including the substantial 1.45 Mb size of the CNV, the absence of similar full-gene gains in large-scale population cohorts (gnomAD-SV and DGV), the supportive computational and clinical recurrence data identified in DECIPHER, and the plausible biochemical mechanism of dosage-dependent kinase hyperactivation described above. In addition to expanding the spectrum of *EIF2AK2*-related disorders, this finding offered potential clinical implications. Early detection of a *de novo EIF2AK2* copy-number gain enabled more accurate prognostic counselling and tailored management. Although no targeted therapies are currently available for *EIF2AK2*-related neurodevelopmental disorders, preliminary preclinical studies have shown that selective PKR inhibition can reverse cognitive deficits and attenuate neurodegeneration in Alzheimer's disease models (28), as well as restore synaptic plasticity (28) and reduce neuroinflammation (29). While these data are currently restricted to experimental models and cannot be directly extrapolated to human neurodevelopmental conditions, they provide a theoretical basis for future research to investigate whether integrated stress response modulators, such as ISRIB or PKR inhibitors, might have potential utility for patients with *EIF2AK2* alterations.

However, our study has limitations. First, functional validation remains to be completed to clarify the underlying molecular mechanisms. Second, the small number of patients currently limits our ability to draw definitive genotype-phenotype correlations. We will continue to gather additional cases and follow up on clinical symptoms and imaging findings. Finally, given the current VUS classification and the non-specific nature of GDD features, we cannot strictly rule out the possibility that the patient's phenotype results from multifactorial etiologies or the cumulative effect of polygenic burden rather than this single structural variant alone. Overall, EIF2AK2 should be regarded as a promising candidate gene whose pathogenic relevance requires further genetic or functional corroboration.

## Conclusion

In this study, we report a complex *de novo* genomic configuration comprising adjacent tetrasomy and trisomy at the 2p22.2 locus involving the *EIF2AK2* gene in a 7-year-old male with GDD. Unlike previously reported pathogenic *EIF2AK2* variants, which are predominantly missense variations, this case suggests that genomic duplication may trigger neurodevelopmental deficits through a dosage-dependent gain-of-function mechanism. The patient exhibited clinical features consistent with *EIF2AK2*-related disorders, including developmental delay, dysarthria, gait disturbances, and hypomyelination. Although formally classified as a Variant of Uncertain Significance (VUS) due to the lack of established dosage-sensitive genes, the specific genomic architecture and strong phenotypic alignment highlight 2p22.2 as a promising candidate locus. Our findings suggest that EIF2AK2 dysregulation likely contributes to GDD regardless of whether it arises from point variations or structural genomic alterations. Ultimately, while this case underscores the potential clinical relevance of *EIF2AK2* copy number gains, in the absence of direct functional validation or further large-scale recurrence data, the causal role of *EIF2AK2* dosage alteration remains a strong but hypothetical candidate mechanism for GDD.

## Data Availability

The original contributions presented in the study are included in the article/Supplementary Material, further inquiries can be directed to the corresponding author.
